# What makes turnips: anatomy, physiology and transcriptome during early stages of its hypocotyl-tuber development

**DOI:** 10.1038/s41438-019-0119-5

**Published:** 2019-03-01

**Authors:** Mengyang Liu, Niccolo Bassetti, Stefan Petrasch, Ningwen Zhang, Johan Bucher, Shuxing Shen, Jianjun Zhao, Guusje Bonnema

**Affiliations:** 1 Plant Breeding, Wageningen University and Research, Wageningen, the Netherlands; 2 Key Laboratory of Vegetable Germplasm Innovation and Utilization of Hebei, Collaborative Innovation Center of Vegetable Industry in Hebei, College of Horticulture, Hebei Agricultural University, Baoding, China; 3 Biosystematics Group, Wageningen University and Research, Wageningen, the Netherlands; 4 Department of Plant Science, University of California, Davis, CA USA

**Keywords:** Plant morphogenesis, Plant breeding

## Abstract

*Brassica* species are characterized by their tremendous intraspecific diversity, exemplified by leafy vegetables, oilseeds, and crops with enlarged inflorescences or above ground storage organs. In contrast to potato tubers that are edible storage organs storing energy as starch and are the vegetative propagation modules, the storage organs of turnips, grown from true seed, are swollen hypocotyls with varying degrees of root and stem that mainly store glucose and fructose. To highlight their anatomical origin, we use the term “hypocotyl-tuber” for these turnip vegetative storage organs. We combined cytological, physiological, genetic and transcriptomic approaches, aiming to identify the initial stages, molecular pathways and regulatory genes for hypocotyl-tuber induction in turnips (*B. rapa* subsp. *rapa*). We first studied the development of the hypocotyl zone of turnip and Pak choi and found that 16 days after sowing (DAS) morphological changes occurred in the xylem which indicated the early tuberization stage. Tissue culture experiments showed a clear effect of auxin on hypocotyl-tuber growth. Differentially expressed genes between 1 and 6 weeks after sowing in turnip hypocotyls, located in genomic regions involved in tuber initiation and/or tuber growth defined by QTL and selective sweeps for tuber formation, were identified as candidate genes that were studied in more detail for their role in hypocotyl-tuber formation. This included a *Bra-FLOR1* paralogue with increased expression 16 DAS, when the hypocotyl starts swelling, suggesting dual roles for duplicated flowering time genes in flowering and hypocotyl-tuber induction. *Bra-CYP735A2* was identified for its possible role in tuber growth *via trans*-zeatin. Weigthed Co-expression Network Analysis (WGCNA) identified 59 modules of co-expressed genes. *Bra-FLOR1* and *Bra-CYP735A2* were grouped in a module that included several genes involved in carbohydrate transport and metabolism, cell-wall growth, auxin regulation and secondary metabolism that serve as starting points to illuminate the transcriptional regulation of hypocotyl-tuber formation and development.

## Turnips: “Neeps” show early differences from non-tuber–forming relative

Turnips show physiological and molecular signs of hypocotyl-tuber development as early as 16 days after sowing, a finding that could help farmers improve the performance of this important food and feed crop. A team led by Guusje Bonnema from Wageningen University and Research in the Netherlands  compared the early anatomy of turnips and another closely related subspecies of *Brassica rapa*, the non-tuber–forming pak choi. They documented differences in the cellular organization of the xylem by day-16 after seed planting. Gene expression profiling between 1–6 weeks after sowing revealed many genes involved in hypocotyl-tuber initiation and growth. These genes affect a range of biological processes, from carbohydrate transport and metabolism to cell-wall growth to hormone regulation. Tissue culture experiments also showed that auxin, a plant growth hormone, promoted early hypocotyl-tuber development.

## Introduction

Storage organ formation is an important developmental process in plants. This process results in formation of tubers, storage roots or corms that both serve as a storage organ for the plant as well as a source of nutrients in food and feed^1^. For the plant, these storage organs provide food storage which enables the plant to survive long dry seasons. In addition, the edible storage organs of many species can be stored easily for long terms and help to provide food security. For this reason, many plant species that form storage organs play significant roles in agriculture. The understanding of the formation of these tubers, storage roots and corms is not only essential to improve agriculture and crop performance but also to generally understand plant development. Tuber formation is best understood in potato where physiologic and genetic studies revealed for instance photo-periodic and hormonal pathways as well as photo assimilates as central components of tuberization^2–6^. In contrast, the underlying genetics of storage organ formation in many other crops remains poorly understood. This poor understanding is mainly due to the diverse nature of the formed storage organs in distantly related plants with often complex genomes that are derived from several different organs.

In contrast to many other crops that form above or below ground storage organs, several *Brassica* species have the unique characteristic that within a single species several morphotypes or crops have been selected that do or do not form vegetative storage organs. This is the case for the four species *B. rapa*, *B. oleracea*, *B. napus* and *B. juncea*^7^. *Brassica* is therefore an excellent model to investigate genetics, evolution and domestication of vegetative storage organ formation. We set out to investigate the development and genetic regulation of the storage organs of turnips (*B. rapa*. ssp. *rapa;* syn. *B. campestris* ssp. *rapifera* L.), which are cultivated as both vegetables and fodder. Like sugar beet and swedes, turnips are grown from true seeds and their above ground storage organs are developed from hypocotyls, with varying amounts of both stem and root tissue^1,8^. Traditionally however, the storage organs of swedes and turnips are referred to as “roots” or “storage roots” as they differ from typical underground tubers, formed from underground stolons, with eyes that sprout to form new plants^9,10^. However, as we set out to understand the genetic regulation, anatomy and physiology of the storage organ formation of turnips, we needed to define from which tissues the storage organs form and name it accordingly. The observation that the storage organs of turnips are swollen hypocotyls with varying degrees of stem and root^1,8^, corresponds with the very smooth skin of the main parts of turnips, devoid of leaf scars or eyes and side roots. However the tops of turnips can display various degrees of leaf scars and develop side shoots. In a previous study QTL for the amount of these side shoots were defined^11^. In addition, adventitious roots can emerge on the bottom of the turnips, and in the worst case leads to so-called “fangy” roots that are unattractive^1^. We decided to name the vegetative storage organs of turnips “hypocotyl-tubers”, to highlight their anatomical origin. Turnips can be divided into Japanese and European turnips that are separated in phylogenetic trees and likely are results of independent domestication^7,8,12^. The two turnip groups differ in many characteristics such as flowering time, leaf shape and response to vernalization. These facts make turnips an especially interesting model for vegetative storage organ formation. The *B. rapa* genome was sequenced already in 2011 and many accessions representing the different morphotypes have been resequenced^13–15^. Analysis of the genome sequence revealed that the *B. rapa* genome, like all *Brassica* genomes, evolved *via* a two-step whole genome triplication and as a result has three syntenic subgenomes^14,16,17^. This genome triplication is hypothesized to have facilitated the diversification of genes as well as gene fractionation and as a consequence led to the evolution of different morphotypes within species but also likely to similar morphotypes between species^18^. In a recent study, *200 B. rapa* accessions and 100 *B. oleracea* accessions representing diverse morphotypes were resequenced and the data were analyzed to identify selection signals for storage organ formation (turnip resp. kohlrabi). This resulted in more than 20 genomic regions under selection in turnip that were enriched for genes involved in cell growth (expansins) and carbohydrate transport^15^.

Another factor that facilitates genomic studies in *B. rapa* (and *Brassica* in general) is the close relationship to radish as well as to the model plant *Arabidopsis* ^14,15,19^. Radish always forms vegetative storage organs and its genome has recently been sequenced^19^. In contrast, *Arabidopsis* does not form vegetative storage organs. Nevertheless, the detailed understanding of pathways and genetics in this model-species can help to understand the genetics and evolution of hypocotyl-tuber formation in *B. rapa* and tuberization in general.

In *B. rapa*, turnip storage organ formation starts with thickening of the region spanning the hypocotyl with various degrees of the taproot and stem^20,21^. The proportion of hypocotyl/taproot that swells differs between turnip accessions^8^. Main mechanism is through secondary growth by a vascular cambium with limited lignification at an early developmental stage^8^.

A number of genetic studies, often phenotyping turnip growth in progeny of crosses between turnips and non-turnip morphotypes, revealed several QTLs, illustrating the quantitative nature of turnip hypocotyl-tuber growth^9,11,22^. Due to the type of genetic markers used these studies can however not be compared to check for shared QTL for turnip formation.

The QTL studies aim at identification of genes that play a role in turnip hypocotyl-tuber growth, and likely will not result in identification of the initial triggers for its storage organ formation. Using detailed cytological observations, comparing hypocotyl growth of turnips and non-tuber forming Pak choi as a control, we previously showed that the initial cellular events that lead to increased radial growth occur between two and three weeks after sowing. We hypothesize that gene expression changes around hypocotyl-tuber initiation lead us to identification of the causal genes.

The aim of this study is to identify the early stages of turnip hypocotyl-tuber growth and effects of auxin on its in vitro growth. We will identify changes in the transcriptome around this stage, and combine this with QTL and selective sweeps for hypocotyl-tuber formation to identify genes with roles in hypocotyl-tuber initiation. Towards this goal we will first phenotype the early stages of hypocotyl-tuber growth by daily sampling hypocotyl tissue and studying the cellular organization in comparison with hypocotyls of non-tuber forming Pak choi, between 14 and 28 days after sowing (DAS). This material will also be used for RNA isolation for quantitative real time PCR (qRT-PCR). Turnip hypocotyl-tuber growth will also be studied in vitro, where we study the effect of auxin on radial growth. In addition, we will select putative genes involved in hypocotyl-tuber formation, based on their expression dynamics quantified using a microarray and their location in QTLs (DH populations derived from crosses between turnip and Wutacai and rapid cycling respectively) and selective sweeps^15^. Transcript abundance of these selected genes will be profiled using the RNA time series of hypocotyl tissues. For a few selected genes, we will present their co-expression modules, based on Weigthed Co-expression Network Analysis (WGCNA) of the microarray data and discuss a number of genes that we extracted as the top 5% genes with the smallest distance based on their expression patterns.

## Materials and methods

### Plant materials and growth conditions

Turnip hypocotyl-tuber anatomy was studied using the DH line VT_013 that we generated from Asian turnip accession CGN06721 (Dutch name: Ronde Rode Heelblad-English name: Scarlet Ball), compared to DH line PC_175 from a non-tuber forming Pak choi accession VO2B0226 (genebank of IVF-CAAS, Chinese name HKG Nai Bai Cai).

One hundred plants per accessions were germinated on 15 February 2017 on filter paper to increase uniformity, and germinated seeds were 22 Feb 2017 transplanted to pots (diameter 17 cm, Lentse potgrond #4). Plants were grown in the greenhouse at Nergena, Wageningen, with temperatures ranging from 14 at night to 28 °C at daytime. For each time point (14, 15, 16, 18, 19, 20, 21, 23, 26 and 28 DAS) three plants (biological replicates) per DH line were used for anatomical sectioning and another set of three plants (biological replicates) were used to harvest RNA.

A doubled haploid Japanese turnip line DH VT_117 from accession CGN 15201 (Toya) was used for a microarray gene expression experiment and this same genotype was also used for in vitro studies. We previously resequenced, assembled and annotated the DH VT_117 genome, and compared it to the *B. rapa* Chinese cabbage Chiifu reference genome^13^. The microarray experimental design consisted of two biological repeats and six time points: hypocotyl tissue was harvested at 7, 14, 21, 28, 35, and 42 DAS. The plants were grown in soil in pots of 17 cm diameter in a climate chamber, with 20 °C day (16 h) and 18 °C night (8 h) temperature. Each biological repeat was obtained by pooling hypocotyl tissue of three different turnip plants. Hypocotyl tissue was removed from the plants and immediately immersed into liquid nitrogen to prevent RNA degradation.

### Auxin treatment in vitro

DH VT_117 and DH PC_175 were selected to investigate the contribution of auxin to hypocotyl-tuber formation. Effects on hypocotyl swelling of addition of the auxin hormone indole-3-butyric acid (IBA) or the two auxin inhibitors 2.3.5-triiodobenzoic acid (TIBA) and N-1-naphthylthalamic acid (NPA), which can inhibit auxin transport, to the media were tested. All the plants were sown in MS-20 (germination medium). Five days after germination seedlings were transplanted to high sugar MS-60 medium with additions of different concentrations of IBA, TIBA and NPA (12 treatments: control, 0.1, 1, 5, 10 μmol IBA; 0.1, 1, 10, 20 μmol TIBA; 1, 10, 20 μmol NPA). For each treatment we tested at least eight replications.

### Anatomy of turnip hypocotyl-tubers

To explore initiation and morphological changes during early tuberization stages of turnip, from six turnip and Pak choi plants the hypocotyls were harvested at defined timepoints and fixed in 4% paraformaldehyde in 0.1 M phosphate buffer (pH 7.2) under vacuum on ice for 30 min and stored overnight at 4 °C. The samples were then washed in the 1× phosphate buffer for 2 × 30 min, and dehydrated in a series of ethanol (30, 40, 50, 60, 70, 80, 90, 100 v/v, 1 h per step) and acetone (100 v/v, 1 h) at 4 °C. Infiltration was carried out using Technovit 8100 basic solution (Heraeus Kulzer, Germany) with hardener I, by transfer of the specimen from acetone to the pre-cooled infiltration solution overnight. We then added hardeners II in reserved infiltration solution to generate polymerization solution at room temperature. Polymerization solution was poured in molds, which are usually used for Technovit embedding, and the specimen was added and immediately covered with transparent PE film for at least 3h. The specimens were blocked with Technovit 3040 (Heraeus Kulzer, Germany). Cross sections of 4 μm were sliced with a Tungsten Carbide knife on a Leica/Reichert 2055 auto cut microtome, stretched on glass dishes with MQ-water and placed on super frost ultra plus microscopy slides. The cross sections were examined under a bright-field microscope (Zeiss Axiophotr, Germany) equipped with a digital camera. Photographs were taken and analyzed using the AxioVison LE Rel.4.6 software (Carl Zeiss, Germany).

### RNA isolation, cDNA synthesis and qRT-PCR analysis

RNA was isolated using the RNeasy® Mini Kit (Qiagen, Germany). RNA quantity and quality were quantified by using a Nanodrop 1000 spectrometer (Thermo Scientific, USA) and by running it on 1% agarose gels respectively. RNA was then stored at −80 °C and reverse transcribed into cDNA using the iScript cDNA Synthesis Kit (Bio-Rad, USA).

*Bra-Actin* and *Bra-Cyp* were used as reference genes for the expression analysis of the selected genes. Expression of *Bra-Actin* was more stable compared to *Bra-Cyp*, calculated by the cycle threshold (Ct) values of each gene at different time points (Supplementary Figure 1).

A set of candidate genes with possible roles in initiation of secondary growth of turnip hypocotyl-tubers was selected from previous data, including QTL, selective sweeps and gene expression data. Two RIL populations were used for QTL mapping: one RIL derived from a cross between a single plant of a vegetable turnip DH VT_115 accession and a late flowering Wutacai accession, while the other RIL was from a cross between a single plant of the same vegetable turnip accession DH VT_115 and a plant of the rapid cycling accession RC_144. QTLs for turnip hypocotyl-tuber traits from these two RIL populations are located on nine chromosomes (1, 2, 3, 4, 6, 7, 8, 9, 10; QTL data are unpublished, marker regions around selected QTL are shown in Supplementary Table S1). Genome regions spanning the selective sweeps for hypocotyl-tuber formation of both turnips and kohlrabi are from our recent publication on the identification of selection signals for hypocotyl-tuber formation by analyzing large resequenced data sets of *B. oleracea* and *B. rapa*^15^. Thirty-one selective sweeps for turnip of varying size on all ten *B. rapa* chromosomes were identified by PiHS^15^.

Combined QTL and selective sweep loci included a large set of 6000 genes. We employed several criteria to select candidate genes. For genes with paralogues, we selected those for which the paralogue displayed a different expression pattern compared to the candidate gene. Furthermore, we selected genes that were differentially expressed in hypocotyl tissue between week 1, 2, 3, 4, 5 and 6 after sowing in DH VT_115 turnip plants (microarray study) and that had different expression patters between the same tissues in a turnip, a Chinese cabbage and a rapid cycling *B. rapa* (RNA-Seq data). From the remaining set of genes, a subset of 58 genes was chosen based on the prediction whether the genes have the potential to be upstream signals (STRING)^23^, or have roles related to storage organ formation based on literature (*Brassica*, potato) (Supplementary Table S2). The subset of 58 so called candidate genes was further screened by profiling their expression using qRT-PCR over hypocotyl and leaf tissue at a single time-point (chosen for its exprssion in the microarray) from two turnip and one Pak choi genotypes^24^. Seven out of the initial 58 genes had a different expression pattern at a defined time point in two turnip accessions compared to the Pak choi accession. The expression of these genes was profiled over the time series of the plants described in this paper (Table 1). Primers of candidate genes were designed by Primer 3.0 software and listed in Supplementary Table S3.

**Table 1:** Details of candidate genes

Gene name	Gene ID	Gene annotation	PiHS	QTL
*EXP3*	Bra024215	Expansin β3	√	
*STP1.1*	Bra019870	Sugar transporter 1.1	√	
*IAA1*	Bra039732	Auxin responsive protein IAA1		√
*SAR1*	Bra010196	Suppressor of auxin resistance	√	
*ARF17*	Bra003665	Auxin response factor 17	√	
*FLOR1*	Bra038700	Floral transition at the meristem	√	√
*CYP735A2*	Bra034022	Cytokinin *trans*-hydroxylase	√	√

PiHS refers to their location in PiHS tuber selected region^14^, QTL refers to their location in QTL region based on the RIL population

The qRT-PCR analysis was performed on a CFX96 Real-Time PCR machine (Bio-Rad, USA) using iQ^TM^ SYBR Green Supermix reagent (Bio-Rad, USA) as a fluorescent detection dye. The qRT-PCR program was performed in 96-well plates under the following protocol: initial activation at 95 °C for 3 min, followed by 40 cycles of 95 °C for 15 s and 60 °C for 30 s. This procedure was followed by melting curve analysis from 95 °C for 10 s, 65 °C for 5 s, and 95 °C for 5 s. The 2^−△△Ct^ method was used to calculate the relative expression levels of the target genes^25^. All reactions were performed on three biological replicates with each two technical replicates.

### Microarray design, annotation and hybridization

A custom microarray based on the two-colour Agilent microarray platform was designed using the reference genome sequence of *B. rapa* cv. Chiifu (Chinese cabbage) v1.0^14,26^. In this custom microarray, 61551 probes were assembled, which represent 40838 *B. rapa* gene IDs. 37713 genes show homology with *Arabidopsis thaliana* genes, while the remaining 3125 are considered unique for *B*. *rapa* (Supplementary Table S4, *B**. rapa* genome was accessed through the database Plant Ensembl Genomes v25 on July 2015). Further, each probe (and the respective genes) were assigned to one of the functional categories of the MapMan onthology, as described in Basnet et al. (2015). The turnip DH VT_117 was grown over 42 DAS in a climate room and sampled at six time points (7, 14, 21, 28, 35 and 42 DAS, see Plant Material) for RNA isolation and cDNA synthesis. This resulted in a total of twelve samples, each time point having two biological replicates. Cy3 and Cy5 dyes were incorporated into cDNA samples following the Agilent Two-Color Microarray-Based Gene Expression Analysis (Low input quick Amp labelling G4140-90050) protocol (Agilent Technologies, USA) and hybridized following a self-self design. Thus, technical replicates of cDNA from each sample were labelled with either Cy3 or Cy5 and hybridized together. In total twelve hybridizations were carried out, one for each sample. Microarray slides were scanned and Cy3 and Cy5 intensities were extracted using the Agilent Feature Extraction software. Raw data were log2 transformed and normalized using R, version 3.4.3. Loess normalization within-array and quantile normalization between-arrays was performed using the *limma* package^27^. Normalized Cy3 and Cy5 values were used for downstream analysis as measures of transcript abundance. Expression of a selection of six genes was validated by qRT-PCR (Supplemental Figure S2).

### WGCNA of microarray data

In order to assess the quality of the twelve samples, the normalized microarray data were analyzed through hierarchical clustering over the samples (Supplementary Figure S3). For each time point, the two biological repeats (R1 and R2) clustered together showing a good quality of the samples. Therefore, the complete dataset was retained for downstream analysis. Normalized transcript abundance values were utilized to build a co-expression network using WGCNA^28^. First, the 20% most variable probes were selected to reduce the computational burden. The resulting dataset of 10280 probes, corresponding to 7654 *B. rapa* genes, was then used to build a step-by-step signed co-expression network. First, a similarity matrix was created by computing the bi-weight mid correlation between all probes. Based on the scale-free topology criterion^29^, an adjacency matrix was created by raising the similarity matrix to the power of 18, resulting in a *R*^2^ = 0.76 for the scale-free fit. The adjacency matrix was then transformed into a Topological Overlap Measure (TOM)-based dissimilarity matrix (DissTOM). DissTOM was used as distance measure for hierarchical clustering of the probes. Modules (clusters of co-expressed genes) were determined using the DynamicTreeCut algorithm with a minimum module size of 50 genes^30^. The overall expression profile for each module can be summarized through its “Module Eigengene” (ME), the first principal component of the expression values across samples for all genes of a module^28^. The modules were further examined to extract co-expressed genes with the candidate genes based on the DissTOM. Thus, smaller DissTOM values indicate that two genes may be potentially co-expressed because: (i) they have similar expression profiles; (ii) they have similar expression profiles to the same set of genes. Accordingly, in order to extract co-expressed genes with the candidate genes, the selected modules were queried for the top 5% genes (“neighbour” genes) with the smallest distance from each of the candidates.

## Results

### Phenotype of hypocotyl of turnip and pak choi during development

**Fig. 1:**
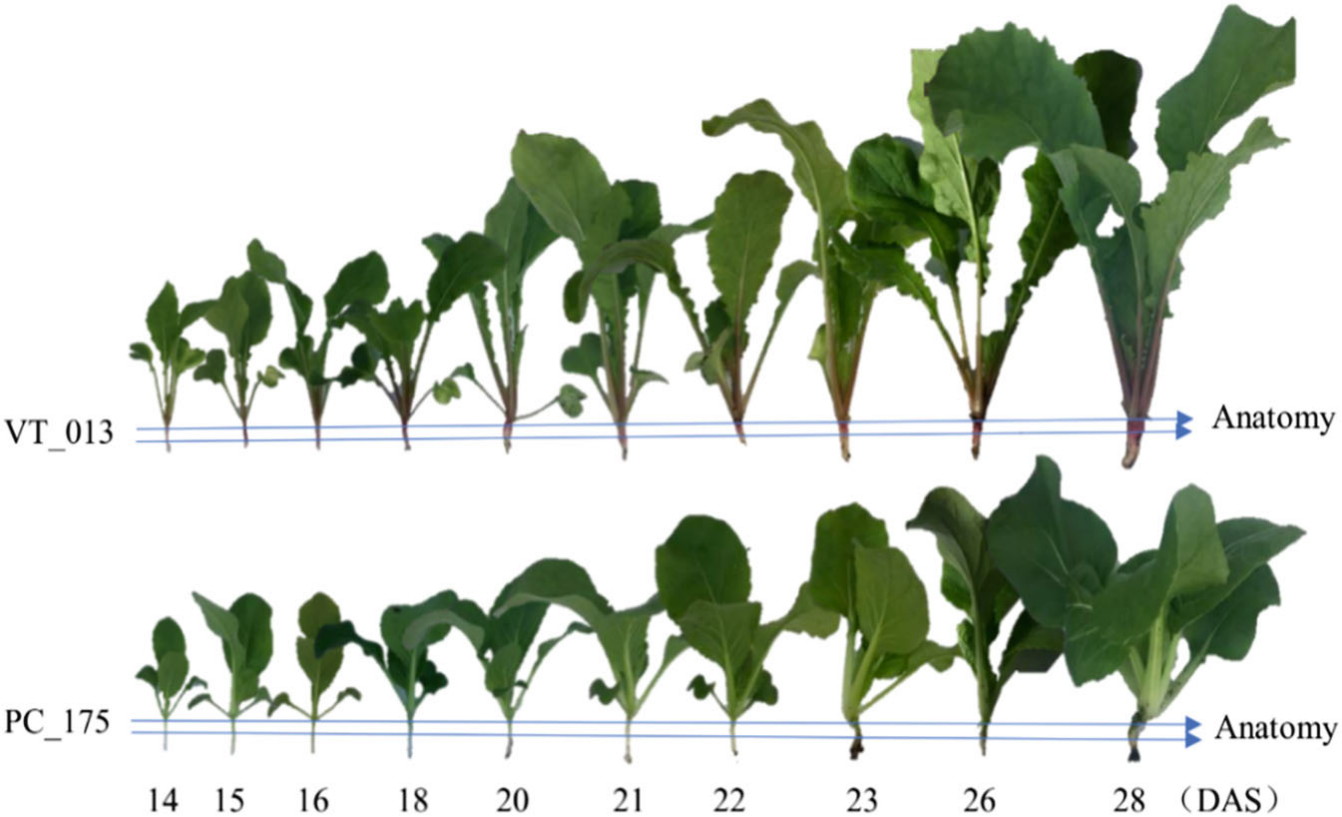
Phenotype of time series of plants of turnip DH VT_013 and Pak choi DH PC_175. DAS indicates days after sowing. Three biological replicates were observed for each timepoint; the figure depicts representative single plants

To reduce variation, six uniform plants were selected per genotype at each time-point (Fig. 1) for anatomical studies and RNA isolation of the hypocotyl tissue. At early stages, till 15 DAS, turnip and Pak choi hypocotyls looked similar when visually observed, except for the color, which was purple in turnip and light green for Pak choi. At 16 DAS, turnip hypocotyls had grown wider than those of Pak choi. At 18 DAS, hypocotyl-tuber onset was clear for the turnip line, even though the hypocotyl of Pak choi also increased in diameter. At 28 DAS the shape of the turnip hypocotyl-tuber emerged in the turnip line. At 42 DAS, the turnip hypocotyl-tuber reached its mature stage (harvest stage); it had a round shape (Supplementary Figure S4).

### Anatomy of hypocotyls of turnip and pak choi

DH VT_013 is an Asian turnip in which the main part of the hypocotyl-tuber develops from hypocotyl tissue. For the anatomical sections we selected the upper part of the hypocotyl, as indicated in Fig. 1.

**Fig. 2:**
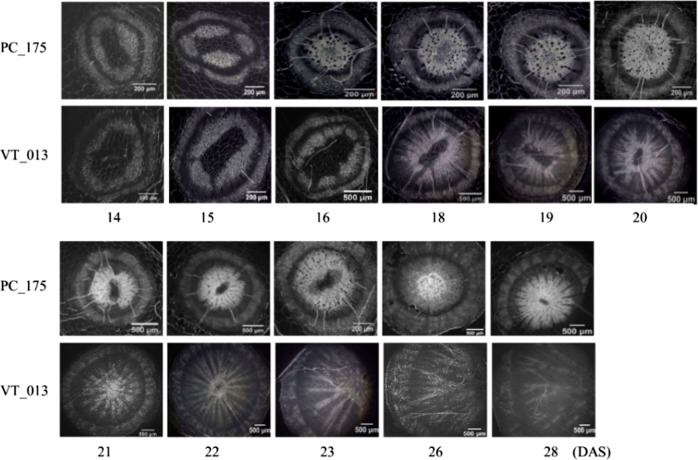
Cross section of upper hypocotyl part of turnip DH VT_013 and Pak choi DH PC_175 at the indicated days after sowing (DAS). Sections were 4 μm thick. Three biological replicates were analyzed at each timepoint; the figure depicts representative single plants

**Fig. 3:**
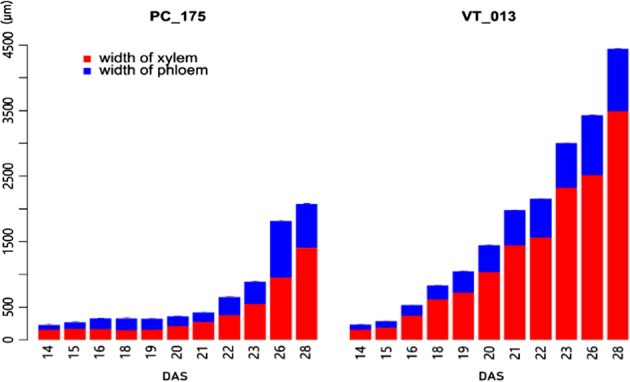
Xylem and phloem width of turnip DH VT_013 and Pak choi DH PC_175 at the indicated days after sowing (DAS). Error bars stand for standard deviation of the three biological replicates

At 14 DAS, cortex, secondary phloem, cambium, xylem and pith can be distinguished clearly, and there are no anatomical differences between the two genotypes. In this stage the cambium is clearly dividing, and the secondary xylem is not circular, but forms four diagonal sections around a large central pith. Growth rate of the different sections was not the same (Figs. 2, 3). At 15 DAS, the xylem in Pak choi hypocotyls appeared slightly lignified, especially cells close to the cambium, while in turnip the width of the xylem ring increased. At 16 DAS the hypocotyl cellular organization of turnip became clearly distinct from that of Pak choi and the diameter of the central part (pith, xylem, cambium, phloem) was twice as large in turnip compared to Pak choi. In Pak choi the xylem cell walls appeared highly lignified and the secondary phloem ring was obviously broadened, while in turnip the cambium circle was active and expanding, while the xylem ring was almost not increased, surrounding a large pith. At 18 DAS we observed clear differences in organization and lignification of xylem between Pak choi and turnip; in turnip hypocotyls the xylem made up the largest part of the cross sections. In Pak choi hypocotyls the xylem was entirely composed of cells with lignified cell walls which resulted in a very dense appearance. In sharp contrast to this, only the xylem vessel cells had thickened cell walls in turnip hypocotyls and these were surrounded by parenchyma cells with thinner cell walls. The lignified vessels were orientated in radial strings from the central pith towards the cambium ring. At 18 DAS the cambium layer together with the secondary phloem were also broader in Pak choi hypocotyls compared to those of turnip. From 18 DAS till 21 DAS, the proportion of xylem in turnip hypocotyls increased indicating further radial growth with clear strings of lignified xylem vessels embedded in xylem parenchyma. In contrast, Pak choi hypocotyls had a strongly lignified xylem layer around the pith. After 21 DAS, Pak choi hypocotyls were still characterized by the lignified xylem layer and showed limited radial growth, while turnip hypocotyls strongly increased in diameter, mainly caused by the rapidly expanding xylem parenchyma layer with radial vessels. As in Pak choi hypocotyls the secondary xylem is characterized by cells with thickened cell walls, which is not the case in turnip hypocotyl secondary xylem with its larger xylem parenchyma cells with thinner cell walls, the boundary between the cambium (meristem and differentiation zone) is clearer in Pak choi. This meristem and differentiation zone is characterized by smaller flat cells with very uniform cell wall thickness. In 28 DAS hypocotyl-tubers of the Japanese turnip DH line VT_117, this layer is slightly thicker (appr. 14 cell layers) than in Pak choi (appr. nine cell layers), which indicates the higher growth rate in turnip hypocotyl-tubers (Supplementary Figure S5). Only minor differences between turnip and Pak choi phloem are observed in their hypocotyls; the phloem appears denser in Pak choi than in turnip.

### Investigation the effects of auxin on turnip hypocotyl-tuber formation in vitro

**Fig. 4:**
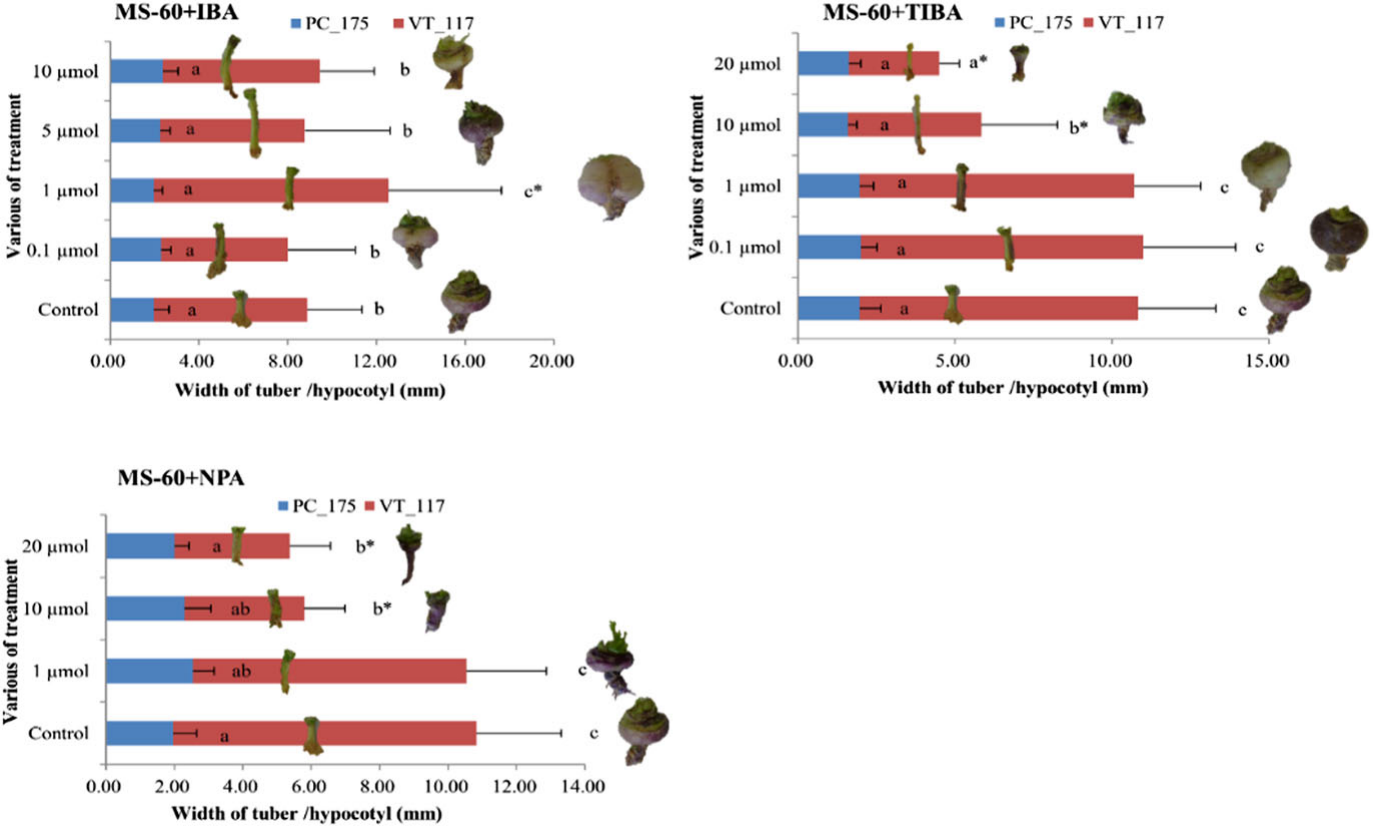
The effect of addition of different concentrations of IBA, TIBA and NPA to MS-60 medium on hypocotyl/ hypocotyl-tuber width on in vitro grown DH VT_013 and DH PC_175. In each diagram the Pak choi hypocotyls are depicted to the left and the turnips are depicted right of the histograms. Error bars stand for standard deviation of the eight biological replicates, * indicates significant difference at *P* < 0.05. If the same lowercase letter appears beside the horizontal bar, values do not differ significantly at *P* < 0.05

In our research, IBA, TIBA and NPA were used to investigate the effects of auxin (Fig. 4). We compared turnip hypocoty-tuber width of in vitro grown plants sown on MS-20 that were transferred to MS-60 with different additions of auxin or auxin transport inhibitors (Supplementary Figure S6). The hypocotyl-tuber width of DH VT_117 at 6 weeks after transfer to MS60 was around 9 mm, and addition of low concentrations of auxin (0.1 μmol IBA) in the medium did not result in a significant change in the hypocotyl width. Addition of 1 μmol IBA resulted in increased diameter, while increased concentrations of IBA (5 and 10 μmol) did not result in increased growth and turnip hypocotyl diameter was comparable to that of the control. In contrast, the width of the Pak choi hypocotyl was not affected by all four concentrations of IBA (0.1, 1, 5, and 10 μmol). Addition of the auxin transport inhibitor TIBA at 10 μmol resulted in significant inhibition of hypocotyl-tuber growth, which was even stronger at 20 μmol TIBA, whereas low concentrations of TIBA (0.1 and 1 μmol) did not affect turnip hypocotyl-tuber radial growth. Addition of 10 and 20 μmol TIBA in the media with Pak choi resulted in a non-significant decrease in hypocotyl-tuber diameter. Similarly to TIBA, addition of 10 and 20 μmol NPA to the MS60 medium significantly inhibited the turnip hypocotyl-tuber growth in DH VT_117, while hypocotyl growth of Pak choi was not affected.

### Gene expression profiling of DH VT_117 turnip hypocotyl tissue

**Fig. 5:**
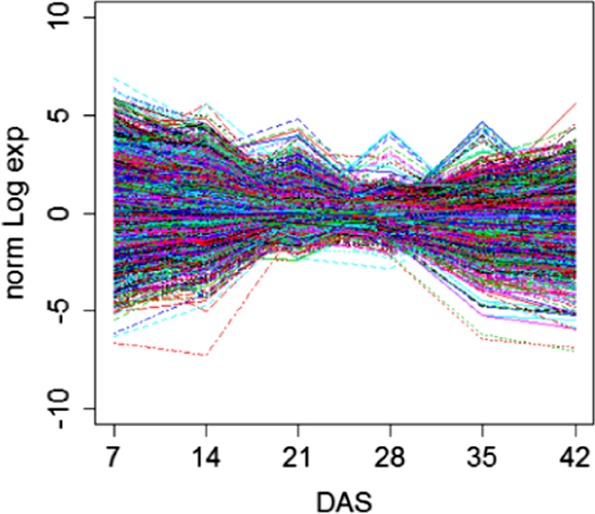
Global overview of the profile of all transcripts on the *B. rapa* custom microarray during turnip development of DH VT_117. Expression values are normalized to the mean to facilitate the visualization. DAS indicates days after sowing

The hybridization profiles of the microarray probes provide a global overview of the transcriptome during the first 6 weeks in DH VT_117 turnip hypocotyls (Supplementary Figure S7). When analyzing the dynamics of transcript abundance, we roughly distinguished two phases (Fig. 5). The wider variation in intensities detected at earlier stages (7 DAS) declines progressively till 21 and 28 DAS (time point 3 and 4). This time frame covers the morphological changes from the germination of the seed until the onset of enlargement of the hypocotyl-tuber (28 DAS). This is followed by an increase in variation until 42 DAS. This second phase corresponds to the increase in diameter of the turnip hypocotyl-tuber through filling with carbohydrates. This global overview of changes in transcript abundance suggests that massive changes in expression profiles of the turnip hypocotyl-tuber occur at the interval between 21-28 DAS, which corresponds to major morphological changes in the turnip hypocotyl-tuber^31^.

**Fig. 6:**
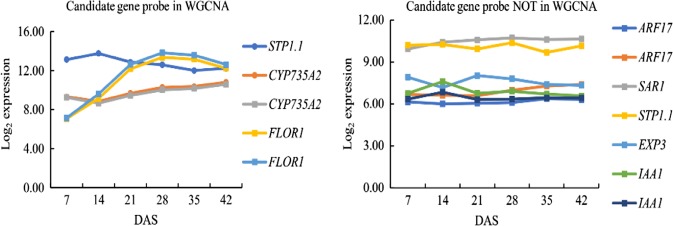
Transcript profiles of candidate genes at 7, 14, 21, 28, 35, 42 days after sowing (DAS). As some genes are represented twice on the microarray, two data sets are plotted

As descried in material and methods, previous studies comparing QTL and selective sweeps for turnip hypocotyl-tuber formation with gene expression profiles and functional information resulted in a selection of seven genes with possible roles in turnip initiation. Their microarray transcript profiles are presented in Fig. 6. *Bra-FLOR1* shows the most evident increase in expression from as early as 7 DAS.

### Gene-expression during the onset of hypocotyl-tuber formation in turnip compared to pak choi

**Fig. 7:**
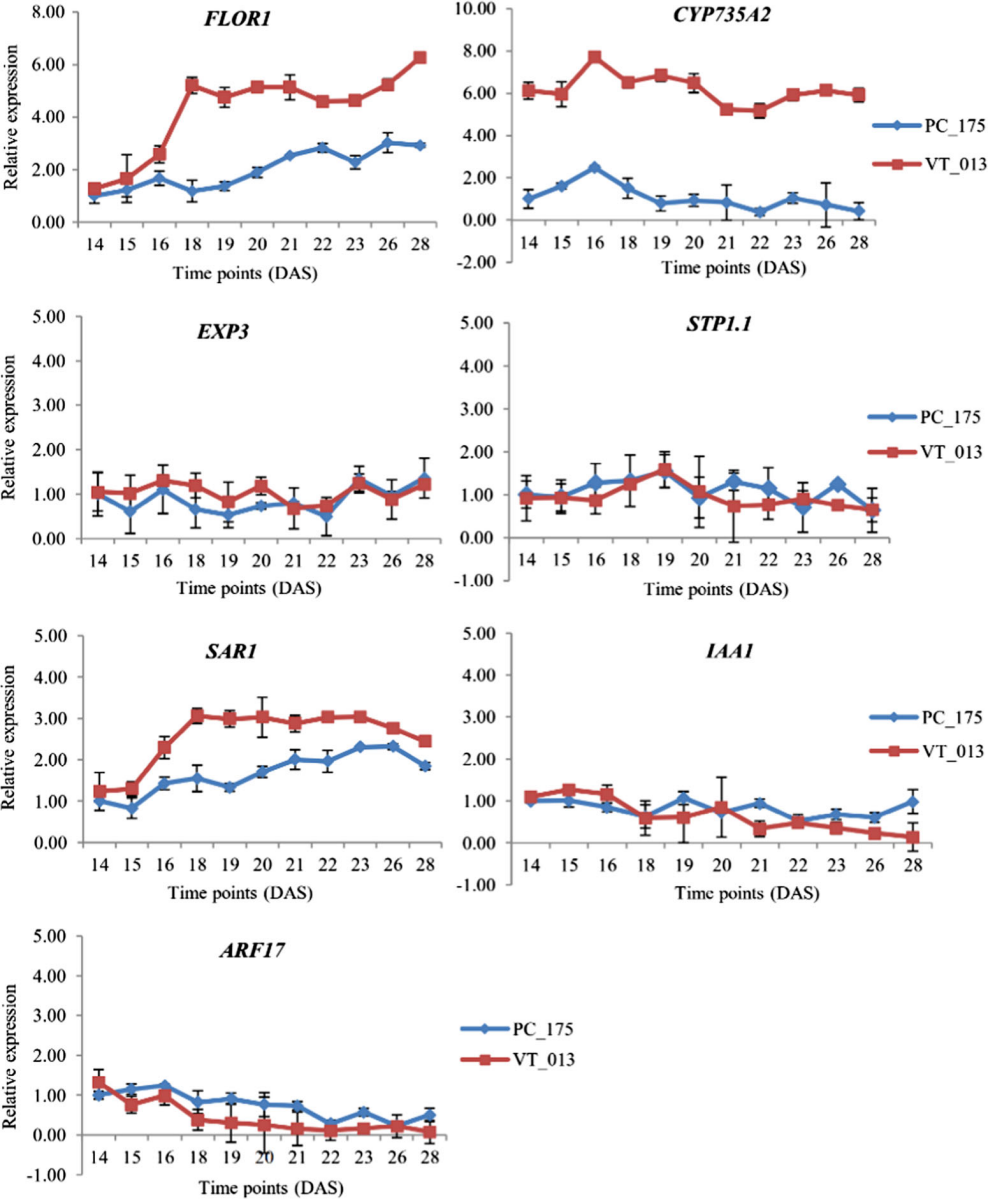
Relative transcript abundance of seven *B. rapa* genes in DH VT_013 and DH PC_175 hypocotyls during plant development. DAS indicates days after sowing. Three biological replicates were analyzed at each timepoint

The expression of the seven genes in turnip and Pak choi at several timepoints between 7 and 28 DAS is presented in Fig. 7. Two candidate genes *Bra*-*FLOR1* (Floral transition at the meristem) and *Bra*-*CYP735A2* (Cytokinin *trans-*hydroxylase) are both located in turnip selective sweeps and hypocotyl-tuber QTLs. In turnip hypocotyls, the expression of *Bra-FLOR1* increased slightly from 14 DAS to 15 DAS, then remarkably increased from16 DAS and reached an expression peak at 18 DAS. Expression remained high (4.59–5.23-fold) after 18 DAS, with an additional increase from 23 DAS till 28 DAS. In contrast with its exprssion in turnip, *Bra*-*FLOR1* transcript abundance in Pak choi hypocotyls increased slowly from 14 DAS, with highest vales of 3.02-fold increase at 28 DAS, which is far below the 6.26-fold increase in turnip tissues. Transcript abundance of *Bra-CYP35A2* was much higher in turnip hypocotyls than in Pak choi hypocotyls at all time points, with a slight fluctuating pattern. The expression level was 5.17–6.85-fold in turnip and 0.42–1.04-fold in Pak choi.

Three genes that are related to the regulation by auxin were profiled for their transcript abundance during hypocotyl development. *SAR1* (Suppressor of auxin resistance 1) and *ARF17* (Auxin response factor 17) were found in selective sweep regions but not in QTLs, *IAA1* (auxin responsive protein IAA1) was located in QTLs, but not in selective sweeps. Contrary to our expectations, the expression of both *Bra-IAA1* and *Bra-ARF17* was not significantly different between turnip and Pak choi hypocotyls; transcript abundance in turnip hypocotyls decreased during hypocotyl-tuber development. On the contrary, the expression of *Bra-SAR1 w*as up-regulated from 15 DAS to 18 DAS in turnip hypocotyls and there after stabilized (2.44-3.06 -fold), while the expression in Pak choi hypocotyls also increased at the same time, with slightly lower transcript levels at all time points. The genes *Bra*-*EXP3* (Expansin B3) and *Bra-STP1.1* (Sugar transport 1.1) were identified in selective sweep regions in both turnip (*B. rapa*) and kohlrabi (*B. olerecea*)^15^. The expression of these two genes showed no obvious difference in turnip hypocotyls compared to Pak choi hypocotyls and the expression did not increase over time during these early stages of hypocotyl-tuber formation and development.

### WGCNA identifies gene modules associated with candidate gene expression patterns

**Fig. 8:**
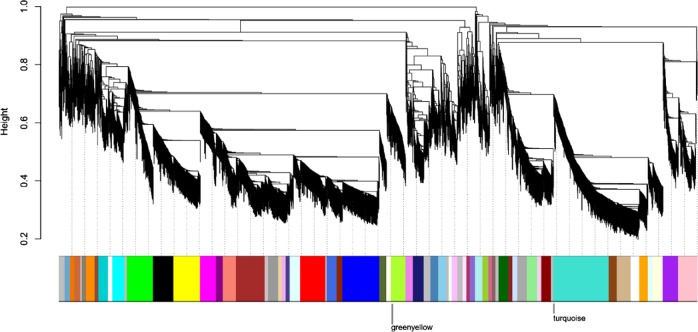
Construction of a signed co-expression network with WGCNA. The dendrogram results from hierarchical clustering on a dissimilarity matrix based on DissTOM distance (see Methods). Branching cut of the dendrogram resulted in 59 co-expression modules (colored bar, each color corresponds to a module/cluster of genes. The modules “turquoise” and “greenyellow” that contained candidate genes are highlighted

**Fig. 9:**
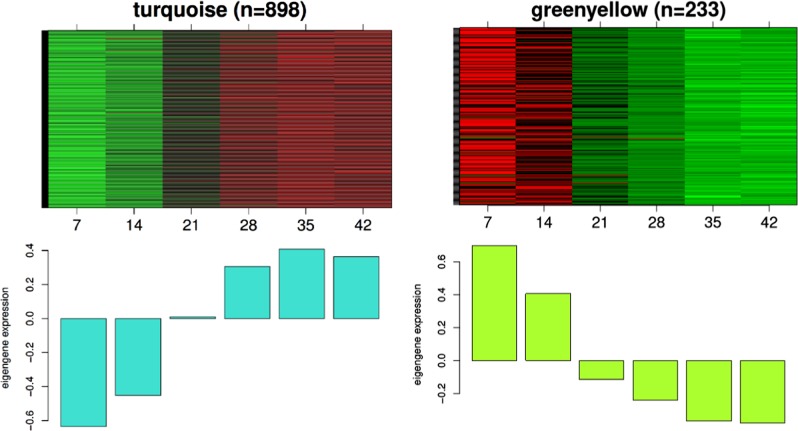
Summary of the modules “turquoise” and “greenyellow” that contained candidate genes. The name of the module is shown on top together with its size (number of probes). Top: Heatmap showing the expression profile of each probe (rows) across seven time points (7, 14, 21, 28, 35, 42 DAS; columns). Bottom. Module Eigengene (ME) summarizing the expression profile of the module

Signed WGCNA grouped the selection of 20% most variable probes into 59 co-expression gene modules, each containing probes with similar transcript abundance in DH VT _117 hypocotyls in a time range from week 1 to week 6 (Fig. 8). Only the genes *Bra-STP1, Bra-FLOR1* and *Bra-CYP735A2* are included in the WGCNA analysis, as the other were not variable enough to be included. The genes *Bra-FLOR1* and *Bra-CYP735A2* are both included in the “turquoise” cluster/module with 898 genes and *Bra-STP1* is in the “green yellow cluster/module with 233 genes. The expression profiles of these clusters are presented in Fig. 9. We set out to identify the genes that are “neighbors” of our candidate genes in these clusters (see Material and Methods). With neighbors, we mean genes that are closer based on the network structure of the clusters and therefore have most similar profile to our candidate genes. The 5% most co-regulated (co-expressed) genes to our candidate gene within the module are presented in Supplementary Table S5.

For *Bra-FLOR1* and *Bra-CYP735A2* in the turquoise module with 898 genes, this resulted in 45 and 45 neighboring genes each, and for *Bra-STP1* in the green yellow cluster with 233 genes this resulted in 12 neighbor genes (Supplementary Table S5). These lists of co-expressed genes were further investigated to see whether it includes genes with obvious roles in turnip hypocotyl-tuber initiation or growth. For *Bra-FLOR1* an interesting co-expressed gene is the gene Bra025774, which is an auxin-responsive family protein (SAUR). In addition, several sugar, carbohydrate metabolism and cell wall genes are co-expressed. For *Bra-CYP735A2* several ABC-transporters, secondary metabolism and cell wall genes are co-expressed and for *Bra-STP1* both carbohydrate metabolism, signaling and secondary metabolism genes are co-expressed.

## Discussion

*B. rapa* includes many crops, also referred to as morphotypes, with extremely different phenotypes. We aim to understand the domestication in *Brassica* species of these extreme phenotypes. Our previous research indicated that the genome triplication shared by all *Brassica* species facilitated independent domestication of these different morphotypes through parallel selection at the subgenome level^15^. The outcome was a set of selective sweeps for turnip and kohlrabi formation enriched for genes involved in sugar or cellulose synthesis and transport, the expansin gene family, root development and cell division. In order to identify candidate genes and their functions in either turnip hypocotyl-tuber initiation or growth, we need to better understand the physiology, anatomy and molecular pathways involved in turnip hypocotyl-tuber formation. As we are especially interested in the initial events of turnip hypocotyl-tuber formation, we first performed anatomical studies to reveal the early developmental stages of turnip hypocotyl-tuber formation and compared this to hypocotyl growth of non-tuber forming Pak choi. This revealed that as early as 16 DAS DH VT_013 turnip hypocotyls had different cellular organization compared to those of Pak choi DH PC_175, specifically through altered expansion and lignification patterns in xylem tissue.

As auxin plays an important role in organ formation and storage organ formation, we studied its effect on hypocotyl-tuber formation. This clearly showed that auxin influences hypocotyl-tuber growth in turnips in vitro, but has no influence on hypocotyl radial growth of non-tuber forming DH PC_175. A microarray study profiling gene expression in turnip hypocotyls during the first 6 weeks of development in combination with selective sweeps and QTL for turnip formation from previous studies resulted in the identification of a small set of genes for which transcript abundance was assessed during initial stages of turnip hypocotyl-tuber growth. A set of co-expressed genes was identified based on this microarray study. The expression data points to a role of the hormone auxin, the flowering time pathway and carbohydrate metabolism and transport in turnip hypocotyl-tuber initiation. Below some details are further discussed.

### Anatomy of turnip hypocotyl compared to pak choi hypocotyl

The anatomy of hypocotyl sections showed clear differences between tuberizing and non-tuberizing morphotypes. The non-tuberizing Pak choi is characterized by a secondary xylem composed of cells with lignified cell walls that resemble the xylem of wood from trees^32^. The secondary xylem of turnip hypocotyl-tubers showed much less cell wall thickening. In the outer part of the xylem ring that is produced by continued secondary growth, only the radial arrays of vessels were composed of cells with thickened cell walls surrounded by cells (likely modified tracheids) with thinner cell walls. The thinner and less lignified cell walls result in the soft tissue that turnips are composed of and make it an attractive vegetable and are likely a result of selective breeding/domestication. The hypocotyl/stem of several *B. rapa* morphotypes, including Pak choi, however, needs to be lignified to be able to carry the harvested product (Pak choi rosette, cabbage head).

Cambium is the secondary meristem that controls the lateral growth of stems and roots. The cambium zone is composed of several cell circles including phloem and xylem mother cells, that may divide several times before differentiating into mature phloem or xylem^33^. A clear characteristic of turnip hypocotyl-tubers is the higher meristematic activity indicated by more layers of cells forming the meristem and differentiation zone in turnip hypocotyl-tubers. The secondary xylem and pith are more irregular and less homogeneous in turnip hypocotyl-tubers compared to Pak choi hypocotyls, which may be caused by the excessive and fast secondary growth (Supplementary Figure S5).

### Exogenous auxin promotes hypocotyl-tuber development of turnips in vitro

Due to its role in many plant developmental processes, a role of auxin in turnip hypocotyl-tuber initiation and growth is expected. In potato a role of auxin in tuber initiation was suggested as auxin levels increase in stolons prior to tuber initiation and remain high during further tuber growth. Auxin also controls the outgrowth of axillary stolon buds similar to above ground shoot branching^34^. Although turnip hypocotyl-tubers are clearly different from potato tubers, mainly because a single turnip forms from the main hypocotyl, contrasting to the many potato tubers formed on stolons, we still expected a role for auxin.

Previous in vitro studies from us and from other groups showed that turnip hypocotyl-tubers will only form in vitro when sucrose concentrations are high^35^ (Supplementary Figure S6). MS-60 with 6% sucrose was chosen as it resulted in reliable formation of hypocotyl-tubers in turnip accessions.

We measured a clear effect on hypocotyl-tuber growth at optimal auxin concentrations (1 μmol IBA), which did not affect hypocotyl growth of Pak choi plants (Fig. 4). In contrast, auxin inhibitors (TIBA and NPA) inhibited hypocotyl-tuber formation of turnips when applied in relatively high concentrations (10 and 20 μmol) and again these inhibitors did not affect hypocotyl growth of Pak choi DH PC_175. This illustrates a clear role for the hormone auxin in turnip hypocotyl-tuber growth.

### Transcriptomic profiling during early hypocotyl-tuber initiation stages

The massive changes in expression profiles at the interval between 21-28 DAS, corresponded to major visible morphological changes like increased growth of the turnip hypocotyl-tuber. A similar globally coordinated change in transcript abundance across major developmental stages has been observed in other tuber forming plants like potato^36,37^, sweet potato (tuberous root)^38^ and radish^39^. However detailed anatomical inspection clearly indicated that developmental changes differentiating turnip hypocotyls from Pak choi hypocotyls occurred already at 16 DAS. This genome wide analysis of the transcriptome during the first 6 weeks of turnip development (Japanese turnip DH VT_117) and presence of differentially expressed genes in previously identified genomic regions (QTL and selective sweeps), was used to select a small set of genes for further investigation. This included profiling their transcript abundance during the very early stages of turnip hypocotyl-tuber development (14 till 28 DAS) and analyses of genes that are co-expressed based on WGCNA analysis of the microarray data (7 till 42 DAS).

Three genes related to auxin (*Bra-SAR1*, *Bra-ARF17*, *Bra-IAA1*) were selected using these criteria. However, as transcript abundance of *Bra-ARF17* and *Bra-IAA1* did not vary between Pak choi and turnip hypocotyls, and only decreased slightly during these early stages of turnip hypocotyl-tuber development, we did not further pursue these genes. Interestingly, miR160 is reported to be up regulated as target of *ARF17* during vegetative storage organ development in radish^40^. The gene *Bra-SAR1*, homologous to the nuclear pore complex (NUP160) in *A. thaliana*, which is suppressor of the auxin resistance gene (*AXR1*)^41^, was up-regulated from 15 to 18 DAS in turnip hypocotyls and displayed a slower increase in Pak choi before stabilizing its expression level. As these three genes were not among the 20% most differentially expressed genes in the microarray, they were not further investigated.

The expression of *Bra-EXPB3* and *Bra-STP1.1*, which was further investigated based on its differential expression between 7 DAS and 42 DAS in the Japanese turnip DH VT_117 and its location in selective sweeps for turnips, did not show significant difference in expression between DH VT_013 and DH PC_175 hypocotyls from 14 till 28 DAS, nor did it change during hypocotyl-tuber development. Expansin proteins are encoded by four groups of genes including α-expansin, β-expansin, expansin-like A, and expansin-like B genes and promote cell wall loosening^42,43^.

The “tubers” of both turnip and kohlrabi are vegetative storage organs formed from hypocotyls and stems respectively, and are rich in carbohydrates (mainly glucose) ^8,15,44^. In mature leaves that act as source tissues, sucrose is synthesized and transported through the phloem to sink tissues, which are both young leaves, roots and storage organs, like for example their tubers^45^. Upon arrival in the sink tissues, sucrose is unloaded from the phloem and transported into the sink cells. Here it can be localized in the apoplast, in the cytoplasm or in the vacuole. The enzyme invertase then cleaves sucrose into glucose and fructose^46^. AtSTP1 and AtSTP13 play important roles in roots during the absorption of monosaccharides from the rhizosphere, and we assume that *Bra-STP1.1* also plays a role in sucrose transport to the growing turnip hypocotyl-tuber^47^.

*Bra-FLOR1* increased sharply in expression between 16 and 18 DAS in turnip hypocotyls only, when hypocotyl-tuber formation starts. *AtFLOR1* encodes a leucine rich repeat protein that promotes flowering in Arabidopsis as it is involved in transition from the vegetative meristem to the floral meristem^48,49^. It is weakly expressed in the vegetative shoot meristem and its protein interacts with the MADS-domain transcription factor AGAMOUS which is strongly expressed in the inflorescence meristem^49^. As the other *Bra-FLOR1* paralogue does not show such an increase in expression, *Bra-FLOR1* is an interesting hypocotyl-tuber initiation candidate gene in turnips for further studies. Dual roles for duplicated flowering time genes in both flowering and tuber induction are well described in the potato system^4,50^ and suggests overlap between tuberization related Gene Regulatory Networks (GRN) with flowering related GRN.

The gene *BrCYP735A2* present in both QTLs for turnip hypocotyl-tuber formation and selective sweeps for turnips, was higher expressed in turnip- than in Pak choi hypocotyls during early time points (from 14 till 28 DAS). In *Arabidopsis*, *AtCYP735A1* and *AtCYP735A2* catalyze the biosynthesis of *trans*-zeatin, which is one of the most common active cytokinins found in higher plants^51,52^. *AtCYP735A2* is mainly expressed in root vascular bundles, as well as in the stem^52,53^. Cytokinin controls cell division and is reported to positively regulate cambium formation and secondary growth^10,54^ and several papers describe that *trans*-zeatin is dispensable for root growth whereas it is involved in regulation of shoot growth, transported *via* the xylem^53,55^. As the turnip hypocotyl-tuber is mainly derived from the hypocotyl, increased *trans*-zeatin levels can indeed explain the hypocotyl-tuber growth in turnip. Also in radish, inductive effects of cytokinins on root cambium growth have been described^10^. Our in vitro experiments testing the effect of cytokinins on turnip hypocotyl-tuber growth were inconclusive (data unpublished). Both genes *Bra-FLOR1* and *Bra-CYP735A2* were in the turquoise module of WGCNA analysis based on their expression during the first six weeks of turnip formation. When inspecting their 45 most neighboring genes based on the WGCNA output, we could identify several interesting genes with potential roles in turnip hypocotyl-tuber initiation. This included carbohydrate metabolism genes and sugar transporters but also genes in cell wall metabolism, signaling genes and a SAUR-like auxin-responsive family protein (Bra025774). In *Arabidopsis*, *AtSAUR53* could promote cell elongation and auxin transport but was not induced by the external application of auxin^56,57^. Over-expression of *SAUR53* (*SAURX53-OX*) exhibited longer roots and hypocotyls. What the role of this gene in turnip hypocotyl-tuber growth is needs further experiments.

Based on the presented results we conclude that detailed anatomical studies of turnip hypocotyl-tuber growth, which indicated that hypocotyl-tuber initiation in the studied genotype started as early as 16 DAS, are important to search for genes involved in turnip hypocotyl-tuber initiation. Tissue culture experiments are important to study the role of phytohormones in turnip hypocotyl-tuber development. Here we showed a clear role for auxin in turnip growth. Global gene expression studies between 7 and 42 DAS in combination with gene expression studies of selected genes at earlier timepoints revealed interesting candidate genes with roles in turnip hypocotyl-tuber initiation.

## Supplementary information

Supplementary materials

Supplementary Tables
