# Supplementary material for: What makes turnips: anatomy, physiology and transcriptome during early stages of its hypocotyl-tuber development
Source: Hortic Res. 2019 Mar 1;6:38. doi: 10.1038/s41438-019-0119-5 (PMC6395767; doi:10.1038/s41438-019-0119-5)
Supplement: Supplementary file 1 — Supplementary materials [file 41438_2019_119_MOESM1_ESM.doc]

**Supplementary**
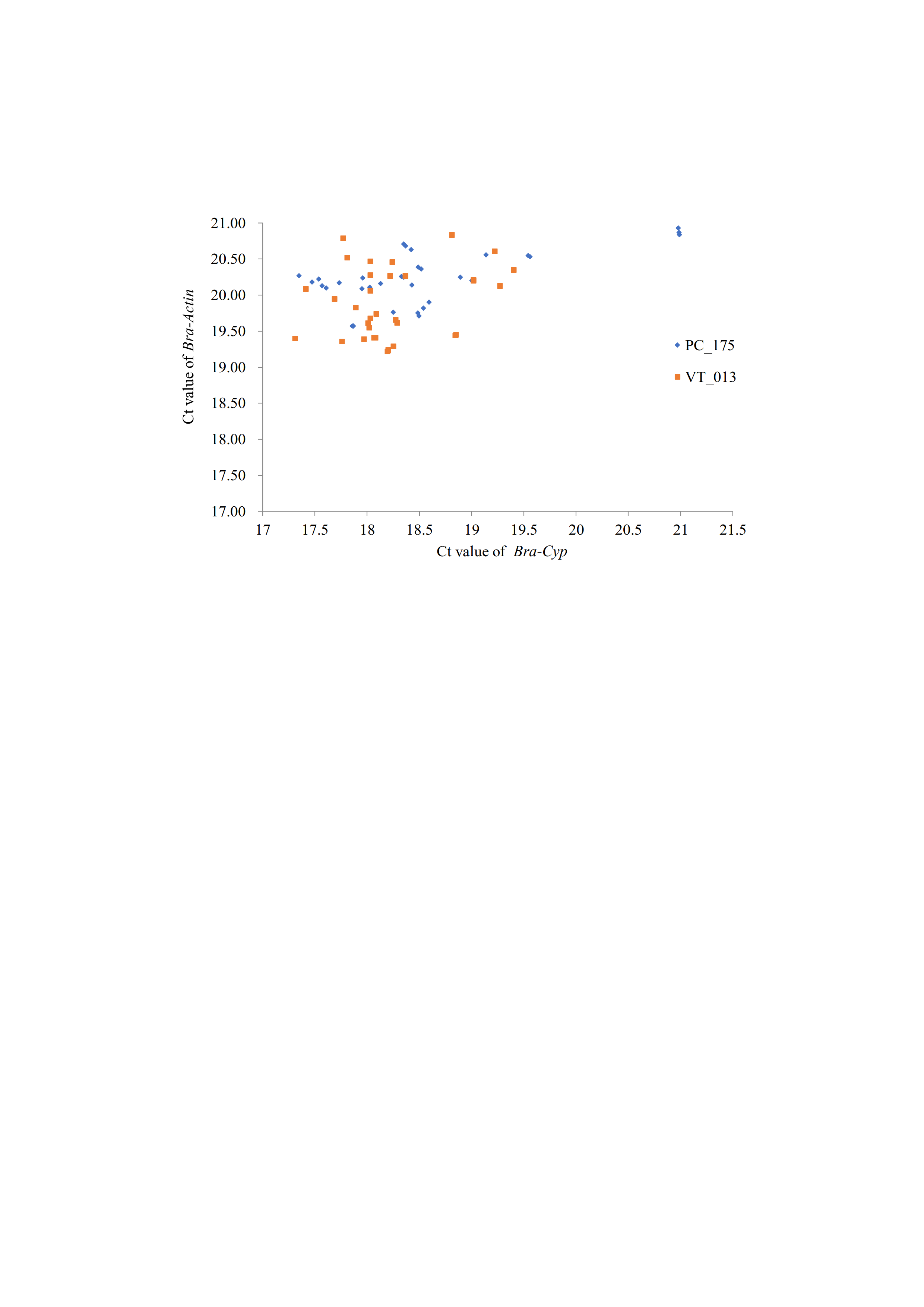
**Figure S1 Ct value of the reference genes *Bra-Actin* and *Bra-Cyp* for all samples.**

d


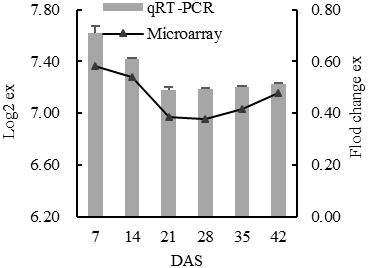

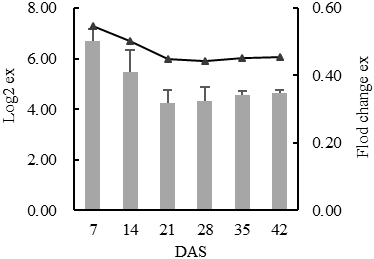

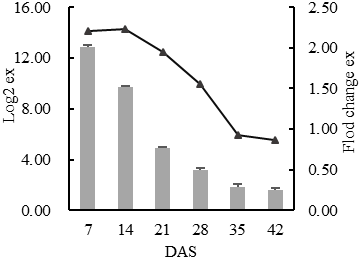

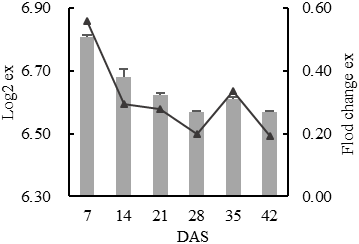

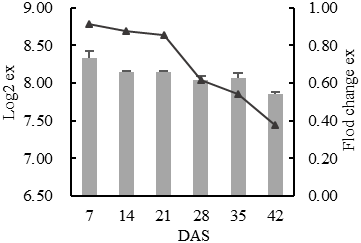

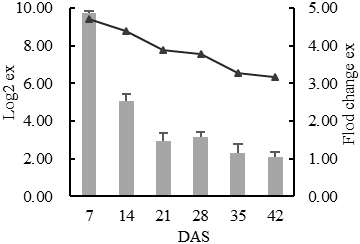


a

c

e

b

f

**Supplementary Figure S2 Validation of microarray data by RT-qPCR.** The gene a-f are presented *Bra-PAB2*(Bra011488), *Bra-MATH1*(Bra035787), *Bra-TF*(Bra020425), *Bra-FMO*(Bra027035), *Bra-SOT16*(Bra008132), *Bra-MATH2*(Bra040904).

**Supplementary**
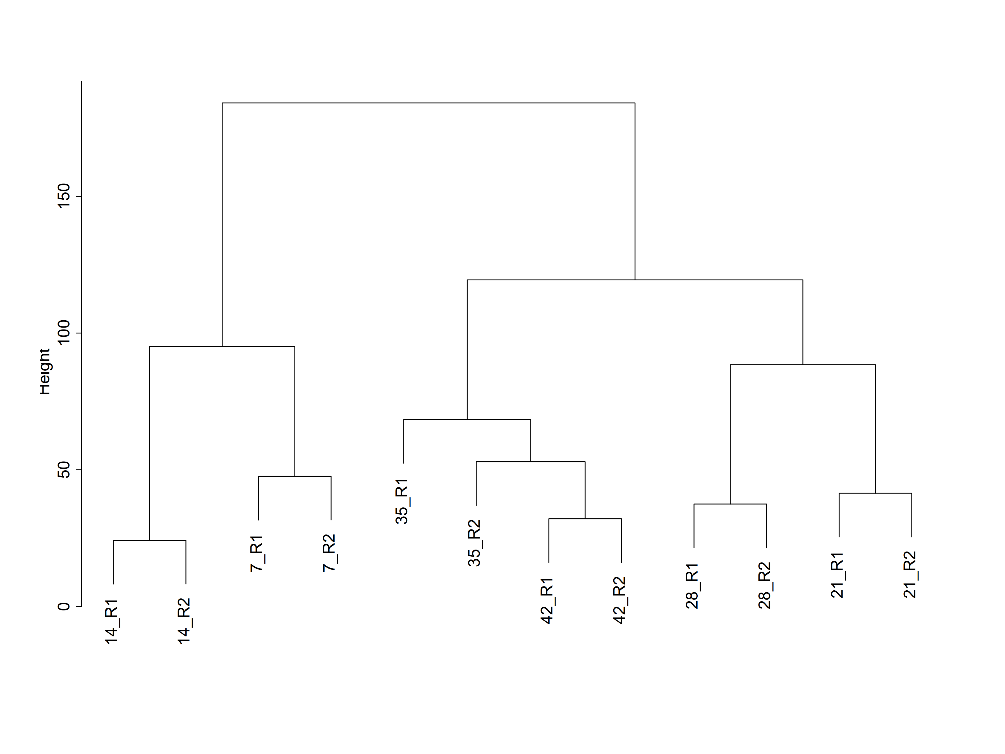
**Figure S3 Hierarchical clustering of *B. rapa* hypocotyl expression patterns across six time points (7, 14, 21, 28, 35 and 42 DAS).** The analysis was carried out on normalized expression values. Each time point is represented by two biological replicates (R1 and R2). Clustering was computed through average linkage clustering based on Euclidean distance. DAS indicates days after sowing.


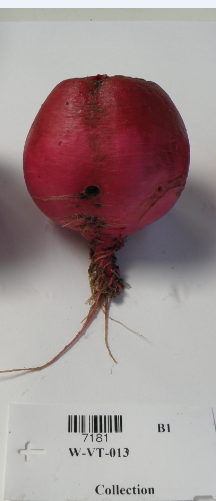


**Supplementary Figure S4 Turnip DH VT_013 in mature stage (42 DAS).** DAS indicates days after sowing.


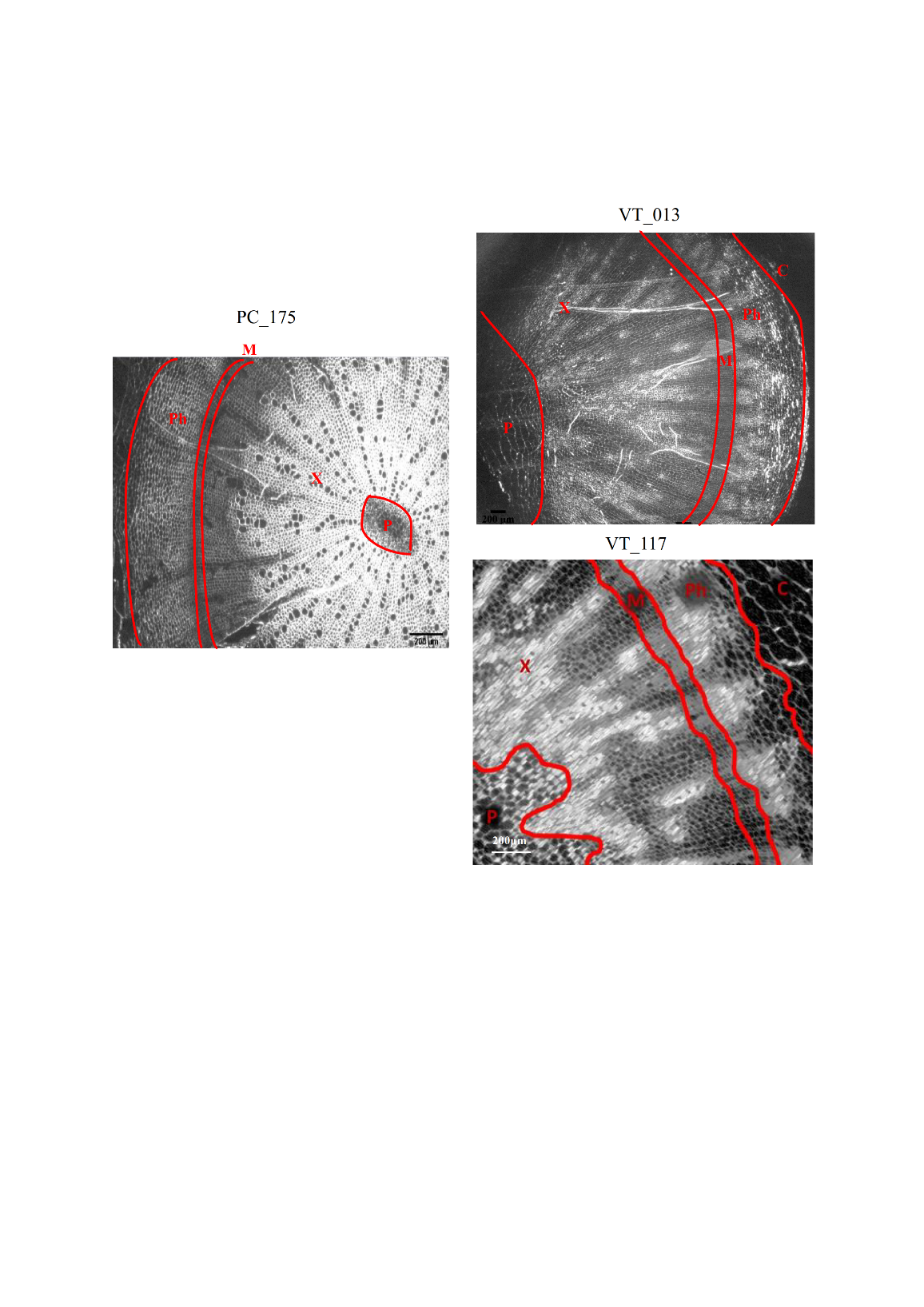


**Supplementary Figure S5 Differences in morphology at 28 DAS Pak choi and turnip hypocotyl.** On the left, a Pak choi (DH PC_175) hypocotyl is shown, whereas on the right two turnip (DH VT_013 and DH VT-117) hypocotyl cross-section is shown. Specific structures are shown: P-Pith, X-(secondary) Xylem, M-Meristem (Cambium), Ph-(secondary) Phloem, C-Cortex. Samples were sectioned to 10 μm.

**Supplementary Figure S6 The hypocotyl-tuber of DH VT_117 in MS-20 and MS-60 *in vitro*. (A)** The hypocotyl-tuber of
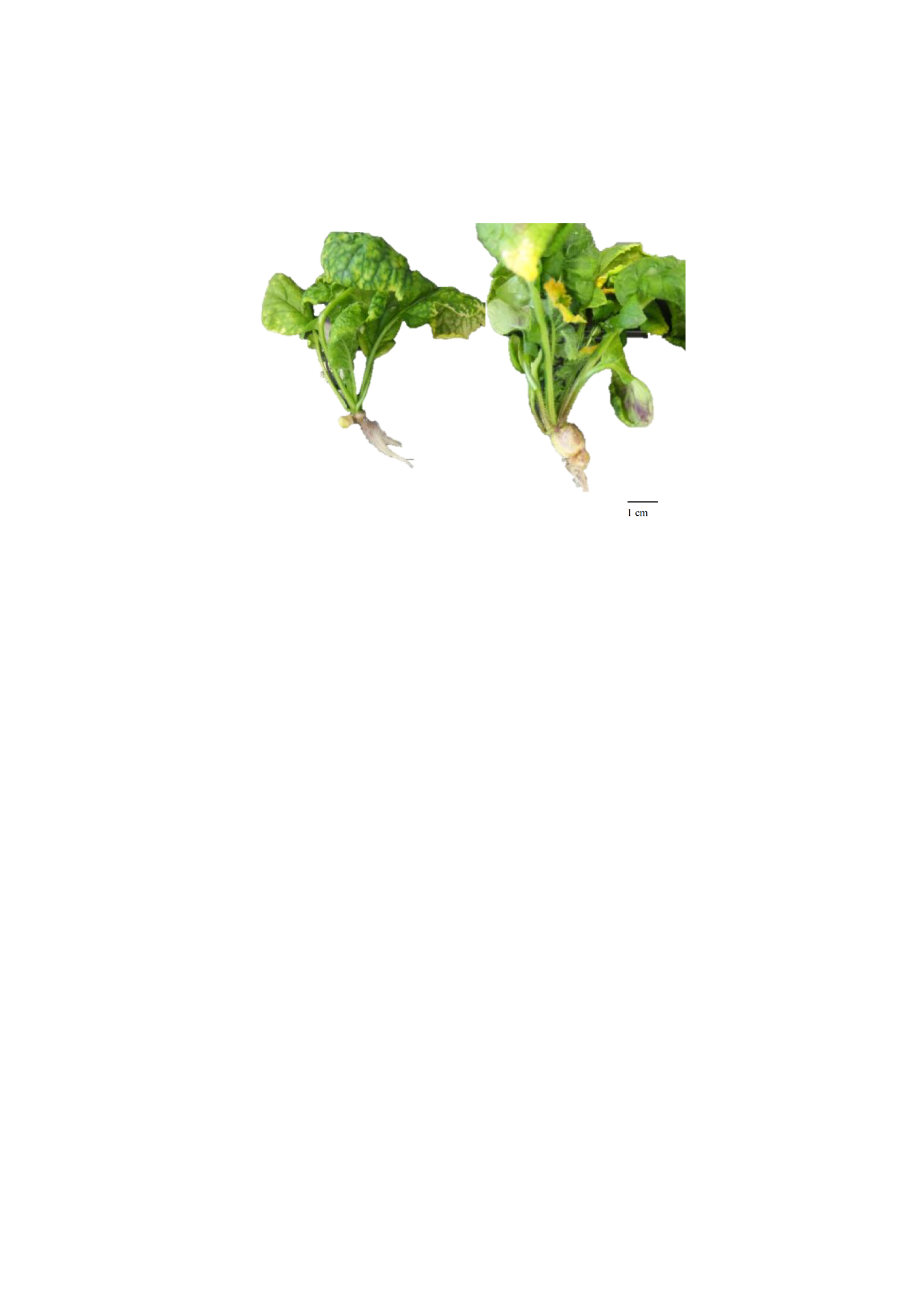
DH VT_117 in MS-20; **(B)** The hypocotyl-tuber of DH VT_117 in MS-60.

**
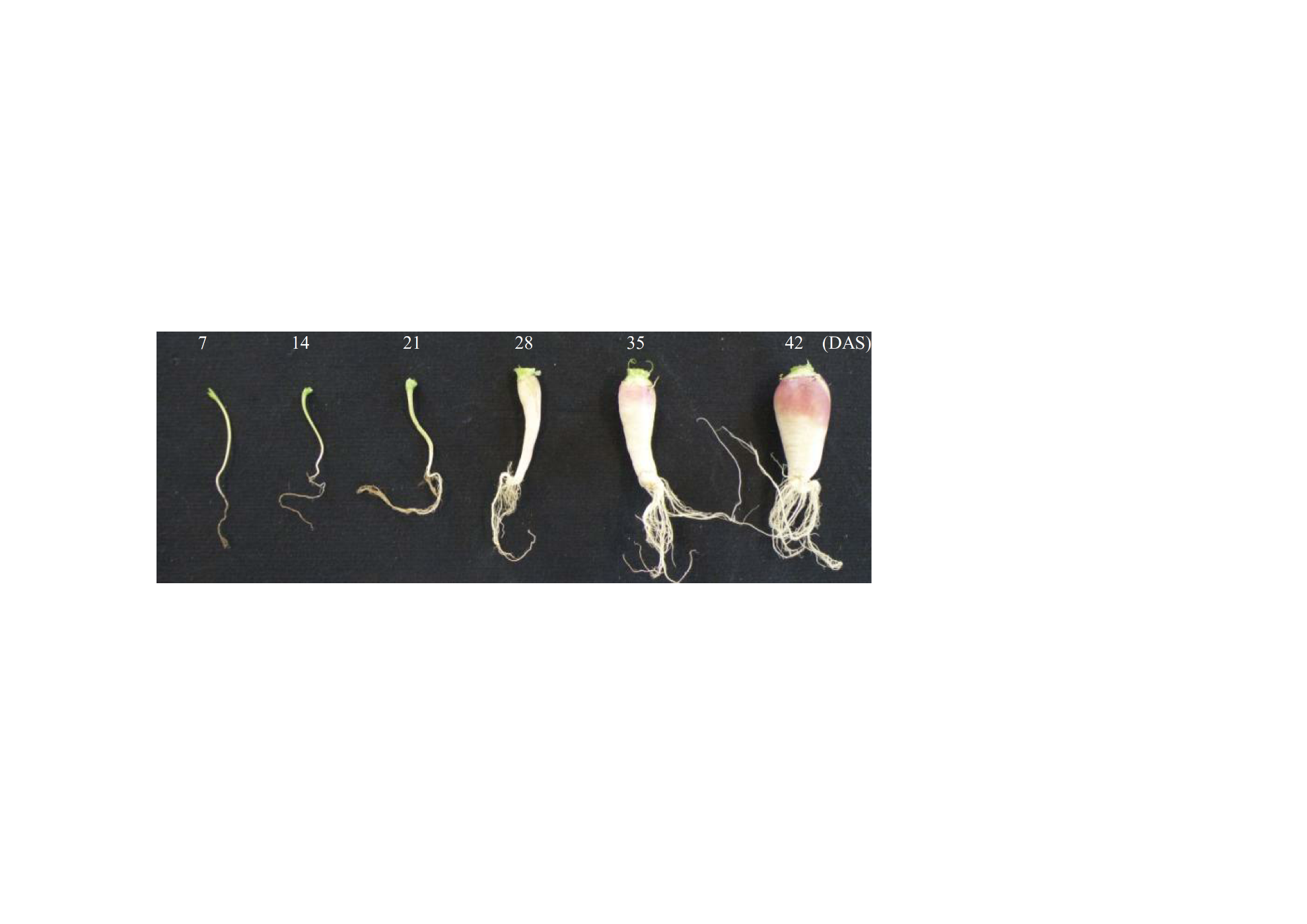
**

**Supplementary Figure S7 Phenotype of DH VT_117** **hypocotyl-tuber at 7, 14, 21, 28, 35, 42 DAS.** DAS indicates days after sowing.
